# Diffuse large B-cell lymphoma in an adolescent female presenting with Epstein-Barr virus-driven hemophagocytic lymphohistiocytosis: a case report

**DOI:** 10.1186/1752-1947-6-141

**Published:** 2012-06-01

**Authors:** Sadaf Altaf, Grace M Atreaga, Avni Y Joshi, Vilmarie Rodriguez

**Affiliations:** 1 Division of Pediatric Hematology/Oncology, Department of Pediatric and Adolescent Medicine, Mayo Clinic, 200 1st Street SW, Rochester, MN 55905, USA; 2 Division Pediatric Critical Care, Department of Pediatric and Adolescent Medicine, Mayo Clinic, 200 1st Street SW, Rochester, MN 55905, USA; 3 Division of Pediatric Allergy and Immunology, Department of Pediatric and Adolescent Medicine, Mayo Clinic, 200 1st Street SW, Rochester, MN 55905, USA

## Abstract

**Introduction:**

Hemophagocytic lymphohistiocytosis is characterized by multisystem inflammation, resulting from prolonged and intense activation of macrophages, histiocytes and CD8+ T-cells. Due to its variable presentation and non-specific findings, timely diagnosis can be challenging. This condition has been associated with malignancies, most commonly with lymphomas and leukemias of T-cell lineage. This case report represents the less commonly associated B-cell lymphomas. We also highlight the difficulties in managing hemophagocytosis with an evolving malignancy. This case report will add to the increasing literature on the diagnosis, complications and management of this complex disorder.

**Case presentation:**

A 15-year-old Caucasian girl, previously diagnosed with Crohn’s disease and treated with 6-mercaptopurine, developed Epstein-Barr virus infection-driven hemophagocytic lymphohistiocytosis. The diagnosis was challenging due to her critical illness and the lack of enough features to fulfill diagnostic criteria at presentation (moderately elevated ferritin, normal coagulation profiles and normal triglycerides). While receiving therapy for hemophagocytic lymphohistiocytosis, she developed bulky cervical lymphadenopathy and was diagnosed with diffuse large B-cell lymphoma. Therapy for lymphoma was initiated and she tolerated the therapy well.

**Conclusion:**

Hemophagocytic lymphohistiocytosis is a rare disorder, but potentially lethal if not diagnosed and treated in a timely manner. Our case highlights the importance of considering this diagnosis in critically ill patients who may not initially fulfill formal diagnostic criteria. In patients diagnosed with hemophagocytic lymphohistiocytosis, occult malignancies should be aggressively ruled out as they can manifest prior to the hemophagocytic lymphohistiocytosis diagnosis or appear during the treatment phase. An accurate diagnosis is also important because management of Epstein-Barr virus-driven hemophagocytic lymphohistiocytosis and Epstein-Barr virus-driven lymphoma differs due to the difference in pathophysiology and the involvement of different immune cell lines.

## Introduction

Hemophagocytic lymphohistiocytosis (HLH) is characterized by multisystem inflammation, resulting from prolonged and intense activation of macrophages, histiocytes and CD8+ T-cells. Minimal clinicopathologic criteria for the diagnosis of HLH, established by the Histiocyte Society [1], include demonstration of a genetic alteration consistent with HLH or demonstration of at least five of the following eight criteria: fever, splenomegaly, cytopenia (affecting two or more cell lines), hypertriglyceridemia, hypofibrinogenemia, hemophagocytosis, low or absent natural killer (NK) cell cytotoxicity, an increased ferritin level and an increased level of soluble interleukin-2 receptor (sCD25) [1].

Two forms of HLH are classically described; primary and secondary. Primary or inherited HLH is an autosomal recessive disorder. Rarely, it can be seen as an X-linked recessive disorder in association with *XIAP* (X-linked inhibitor of apoptosis protein, formerly *BIRC4*) mutations and X-linked lymphoproliferative disease. It usually manifests during infancy, but rare cases have been described in older children and adults [2]. The secondary or acquired form is caused by a variety of diseases including viral, bacterial and protozoan infections, malignancies and macrophage activation syndrome associated with autoimmune disorders [3].

## Case presentation

A 15-year-old Caucasian girl with Crohn’s disease, who was on immunosuppressive therapy with oral 6-mercaptopurine, presented with fevers and sore throat, six months after her diagnosis of inflammatory bowel disease. On examination, she had bilateral tonsillar hypertrophy with basilar exudate. Epstein-Barr virus (EBV) serologies showed the presence of viral capsid antigen immunoglobulin M, immunoglobulin G and positivity for EBV nuclear antigen suggesting reactivation of the infection (Table 1). She went on to develop significant jaundice and liver function tests revealed elevated transaminases and direct hyperbilirubinemia (Table 2). An ultrasound of her liver did not show any obstructive pathology. The patient was hospitalized because of her persistent fevers along with jaundice, with the admitting diagnosis of EBV-induced cholestatic hepatitis. Serologies for hepatitis A, B and C, cytomegalovirus and human immunodeficiency virus (HIV) were negative. EBV copies continued to rise throughout the course of her illness, from 1,500 to 20,000 copies/mL. She continued to be febrile and developed severe respiratory distress and hypoxemia, requiring transfer to the pediatric Intensive Care Unit.

**Table 1:** Epstein-Barr virus serologies during the course of illness

	**Diagnosis of HLH**	**At completion of therapy**
EBV nuclear antigen	Positive	Negative
EBV viral capsid antigen immunoglobulin G	Positive	Negative
EBV viral capsid antigen immunoglobulin M	Positive	Negative

EBV: Epstein-Barr virus; HLH: hemophagocytic lymphohistiocytosis.

**Table 2:** Laboratory data at diagnosis of hemophagocytic lymphohistiocytosis and lymphoma

	**Reference range**	**Diagnosis of HLH**	**Diagnosis of lymphoma**
Hemoglobin (g/dL)	12 to 15.5	7.7	9.6
White blood cells (×10^9^/L)	3.5 to 10.5	0.3	3.1
Absolute neutrophil count (×10^9^/L)	1.7 to 7.0	0	2.39
Platelets (×10^9^/L)	150 to 450	17	99
Ferritin (mcg/L)	11 to 307	4,485	1,749
Fibrinogen (mg/dl)	200 to 430	309	
Aspartate transaminase (U/L)	8 to 43	146	38
Alanine transaminase (U/L)	7 to 45	264	45
Total bilirubin (mg/dL)	0.1 to 1.0	6.5	0.7
Direct bilirubin (mg/dL)	0.0 to 0.3	4.4	0.4
Triglycerides (mg/dL)	-	341	-
Epstein-Barr virus copies	-	20,500	1,500
Soluble CD25 (U/mL)	45 to 1105	39,322	-

HLH: hemophagocytic lymphohistiocytosis.

**Figure 1:**
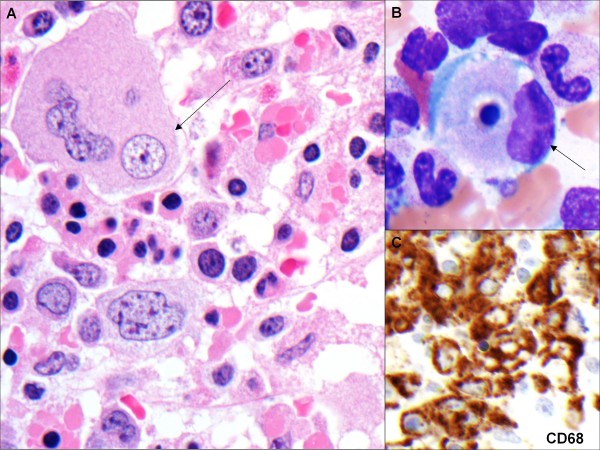
Bone marrow biopsy and aspirate showing hemophagocytosis and positive staining for CD68 marker for the presence of macrophages.

A bone marrow biopsy and a diagnostic lumbar puncture were performed. Her cerebrospinal fluid showed evidence of pleocytosis. The bone marrow biopsy showed pancytopenia and increased lymphohistiocytic infiltrates with prominent hemophagocytosis (Figure 1). The significant inflammatory response with high fevers and pancytopenia was attributed to her being infected with EBV. A decision was made to administer one dose of rituximab and high dose immunoglobulins to control the exaggerated immune response.

There was laboratory evidence of cytopenia, with results notable for falling neutrophil count, severe anemia and thrombocytopenia (Table 2). There was moderate elevation of ferritin and triglycerides. Her soluble CD25 (soluble interleukin-2 receptor) level was elevated at 39,322U/mL. (this information was not available at initial presentation). There was complete absence of CD16 and CD56 positive NK cells. NK cytotoxicity, porphyrin and granzyme testing could not be done with the initial presentation due to the absence of NK cells. Fibrinogen and coagulation studies remained within normal limits. Our patient was diagnosed with EBV-driven HLH as she fulfilled five of the eight required criteria for diagnosis, and she was started on chemotherapy with etoposide, dexamethasone and ciclosporin based on HLH 2004 protocol.

Testing was also done to rule out HIV and other immune deficiencies. Genetic testing was carried out to rule out *PRF 1*, *UNC13D, STX11* and *STXBP2* mutations as well as signaling lymphocytic activation molecule-associated protein expression (SLAM) on cytotoxic lymphocytes.

Our patient had moderate cervical lymphadenopathy (2cm), which was attributed to her EBV viremia at the time of diagnosis of HLH. There was moderate splenomegaly on a computed tomography scan of her abdomen but no other significant lymphadenopathy was noted, hence the diagnosis of lymphoma was not strongly considered. No further doses of rituximab were administered once HLH therapy was started. Her chemotherapeutic regimen included dexamethasone, etoposide and ciclosporin. Our patient received eight weeks of therapy on this protocol. She responded to therapy and improved clinically.

**Figure 2:**
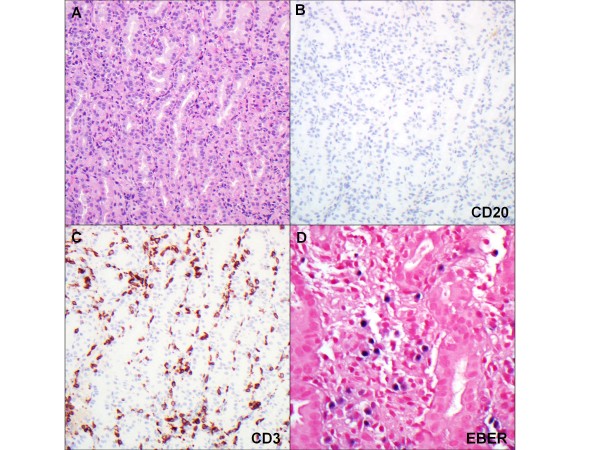
**Lymph node biopsy showing atypical diffuse large B-cell lymphoma cells positive for CD20, immunohistochemistry showing negativity for CD3, and** ***in situ*** **hybridization using Epstein-Barr virus encoded probes showing diffuse positivity for the virus.**

**Figure 3:**
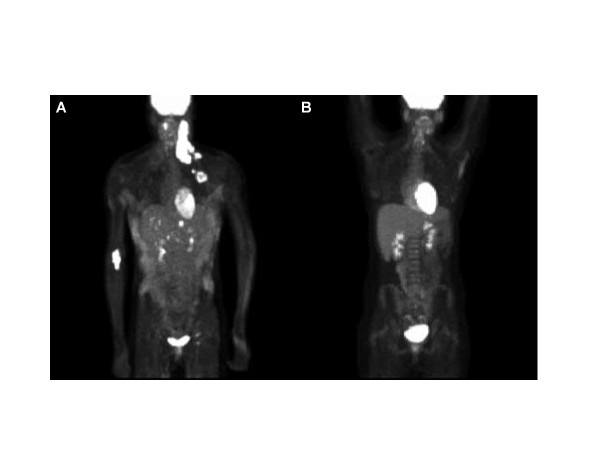
Positron emission tomography scan at diagnosis of lymphoma and at completion of therapy.

Due to progressive enlargement of the lymph nodes in her cervical region two months after the initiation of therapy, an excisional biopsy of the cervical mass was performed (Figure 2). Pathology results were consistent with EBV-positive diffuse large B-cell lymphoma. EBV viremia at the time of diagnosis of lymphoma was reported at 1,500 copies in the blood (Table 2). Immunohistochemistry results were positive for CD20, CD19 and CD79a. A positron emission tomography scan showed extensive involvement of her supraclavicular and cervical lymph nodes as well as some involvement of her liver (Figure 3). A bone marrow biopsy showed no evidence of involvement of lymphoma or hemophagocytosis. Treatment for lymphoma was given with six cycles of rituximab, cyclophosphamide, doxorubicin, vincristine and prednisone (R-CHOP) based on our institutional standard of care for patients sixteen years of age and older. Treatment related complications included prolonged fever and neutropenia. Restaging with positron emission tomography at the end of her R-CHOP therapy showed complete resolution. Our patient is now disease-free after completion of therapy. She has been off immunosuppressives for her Crohn’s disease since her recovery from lymphoma, and has also been in remission for over 24 months now.

## Discussion

This case highlights the importance of making the diagnosis of HLH in a critically ill patient. The challenge presented in our patient was the limited number of HLH diagnostic criteria, with only a moderate elevation of ferritin noted compared with other patients diagnosed with HLH [4]. There was absence of a coagulopathy and abnormal triglycerides. Hence, at her initial presentation, she did not fulfill five of the eight criteria. Eventually she fulfilled the required five of eight criteria, including persistent fevers, cytopenia, splenomegaly, elevated CD25 and presence of hemophagocytes on a bone marrow biopsy. She had elevated EBV serologies and polymerase chain reaction copies.

Infection-associated HLH has been reported with EBV, cytomegalovirus, hepatitis A, B and C, parvovirus, human herpes virus 6 and 8, HIV, measles, mumps and rubella. In addition, a variety of bacterial, fungal and protozoan infections have been described [5].

Patients with inflammatory bowel disease on immunomodulating drugs like azathioprine and 6-mercaptopurine have been reported to develop HLH [6,7]. The diagnosis of our patient’s Crohn’s disease was confirmed by biopsy showing evidence of terminal ileitis. She was on immunosuppressive therapy for six months with 75mg of 6-mercaptopurine prior to the diagnosis of HLH. Further extensive evaluation for immune deficiencies did not reveal any associated primary immunodeficiency to be the cause.

In our patient, there was no clear evidence of lymphoma at presentation, with an absence of significant lymphadenopathy on clinical examination and imaging (computed tomography of her chest, abdomen and pelvis). Physical and imaging studies suggested the possibility of reactive lymphadenopathy due to EBV viremia and an excisional biopsy was not pursued at initial presentation. It is possible that our patient might have presented with smoldering lymphoma at the time of HLH presentation. Immunosuppression controlled the cytokine storm secondary to EBV; however, our patient had clear clinical and imaging evidence of lymphoma by the time she completed the induction phase for her HLH.

Malignancy-associated HLH may develop before the diagnosis of cancer or during the treatment phase. It is mostly described in association with T-cell or NK cell lymphomas or leukemias [8-10]. Hemophagocytosis and B-cell lymphomas are described in the majority of Asian patients and it is hypothesized that there may be genetic and environmental factors pertinent to this population which may be responsible for the transformation to B-cell lymphoma [11]. Hence, it is recommended that, in children with systemic HLH, a diagnosis of non-Hodgkin lymphoma should be aggressively pursued if there is suspicion about the possible presence of a malignancy [12].

Defects in interleukin-2 inducible T-cell kinase gene *(ITK)* are being noticed in females with EBV-induced HLH, clinically resembling X-linked lymphoproliferative disorder [13]. Testing for ITK did not show any abnormality in the gene. Over 90% of patients with defects in the *XIAP* gene develop HLH, associated with and without EBV. These patients are also at risk for recurrent HLH [14]. X-linked recessive inheritance of *XIAP*, is rarely seen in females, unless there is skewed inactivation of the wild type X chromosome. We have not completely ruled out this rare possibility in our patient. HLH is a disorder with an evolving spectrum of presentation. So far, 70% of the patients with familial HLH have an identified genetic mutation; 30% of North American patients do not have identified mutations in any of the four relevant genes [15]. As more genetic associations are identified, we will learn more about genotype-phenotype correlations in this disorder.

Our report highlights the spectrum of findings associated with EBV infections, and the clinical overlap between EBV-driven HLH and EBV-driven lymphoma. The initial presentation was that of secondary HLH related to EBV infection, which was suspected due to persistently rising EBV titers and the possibility of a weakened T-cell system from the immunosuppressive therapy for Crohn’s disease. Our patient may have been in the pre-clinical stages of lymphoma triggered by EBV affecting the B-lymphocytes, and this transformation was possibly hastened by the presence of impaired T-cells and histiocytes. The clinical distinction between EBV-related HLH and EBV-associated lymphoma is also important for management purposes. Our patient responded clinically to HLH therapy, but would have gone on to develop further complications of lymphoma if this had not been promptly recognized. After completion of the lymphoma therapy, our patient has been in complete remission. Due to a lack of evidence of a primary immune deficiency and excellent response to therapy, bone marrow transplantation was not considered. Vigilant follow-up with imaging at scheduled intervals will be required for a prolonged period of time.

## Conclusion

HLH is a rare disorder, but potentially lethal if not diagnosed and treated in a timely manner. Our case highlights the importance of considering this diagnosis in critically ill patients who may not initially fulfill formal diagnostic criteria. Potential complications of this disorder, including malignancy, should also be anticipated. In patients diagnosed with HLH, occult malignancies should be aggressively ruled out.

## Consent

Written informed consent was obtained from the patient for the publication of this case report and any accompanying images. A copy of the written consent is available for review by the Editor-in-Chief of this journal.

## Competing interests

The authors declare that they have no competing interests.

## Authors’ contributions

SA compiled and analyzed the patient’s data and wrote the initial draft. GA assisted with the critical illness part of the draft. AJ edited the immunological aspect of the draft. VR reviewed the final manuscript and edited the oncological aspect of the draft. All authors read and approved the final manuscript.
